# Editorial: Physical Activity, Sedentary Behaviors and Exercise Interventions in Children

**DOI:** 10.3390/sports14100462

**Published:** 2026-10-19

**Authors:** Pedro Duarte-Mendes, Daniel A. Marinho, Fernanda M. Silva

**Affiliations:** 1 Department of Sports and Well Being, Polytechnic University of Castelo Branco, 6000-084 Castelo Branco, Portugal; 2 Sport Physical Activity and Health Research & Innovation Center (SPRINT), 6000-084 Castelo Branco, Portugal; 3 Department of Sport Sciences, University of Beira Interior, 6201-001 Covilhã, Portugal; 4 Research Center in Sports Sciences, Health Sciences, and Human Development (CIDESD), 6201-001 Covilhã, Portugal; 5 School of Education and Communication, Universidade do Algarve, 8005-139 Faro, Portugal; 6 CIPER, Faculty of Sport Sciences and Physical Education, University of Coimbra, 3040-248 Coimbra, Portugal; 7 Sport Physical Activity and Health Research & Innovation Center (SPRINT), 8005-139 Faro, Portugal

## 1. Introduction

Childhood represents a critical developmental period for establishing behaviors that shape health trajectories across the lifespan [1]. Recognizing the importance of physical activity for healthy growth and development, the World Health Organization (WHO) recommends that children and adolescents aged 5–17 years accumulate at least 60 min of moderate-to-vigorous physical activity (MVPA) daily while limiting sedentary behavior [2].

However, meeting these recommendations remains a major public health challenge. Global surveillance data indicate that a substantial proportion of children and adolescents do not achieve the recommended levels of MVPA [3], while sedentary behavior remains highly prevalent worldwide [4]. This is particularly relevant given the well-established role of regular physical activity in supporting child and adolescent health, with consistent evidence demonstrating benefits for physical fitness and body composition [5,6], motor competence [7], and cognitive and psychosocial well-being [8]. Conversely, sedentary behavior, particularly recreational screen time, is associated with adverse health outcomes, including lower physical fitness, poorer cardiometabolic health, shorter sleep duration, unfavorable measures of adiposity, and poorer mental health [9,10]. In parallel, recent global projections suggest that the prevalence of childhood overweight and obesity may increase substantially over the coming decades, emphasizing the urgent need for comprehensive strategies to promote healthy lifestyles from an early age [11].

Addressing these challenges requires research that advances understanding of the factors associated with child and adolescent health and generates knowledge relevant to active and healthy lifestyles. Integrating knowledge on physical activity, sedentary behavior, physical fitness, nutrition, and exercise is particularly important for informing public health, educational, and sport-related practices throughout childhood and adolescence.

Against this background, this Special Issue was launched to provide new evidence on different dimensions of child and adolescent health and development. The five articles included in this first volume address motor competence and physical development; the relationships of physical activity, sedentary behavior, and physical fitness with body composition; associations between nutritional behaviors, body mass index, and physical performance; and the planned synthesis of evidence on the effects of exercise on executive function. Collectively, they provide cross-sectional and longitudinal evidence, alongside a protocol for future evidence synthesis, addressing gaps related to motor development, body composition, lifestyle behaviors, physical performance, and cognitive health.

## 2. An Overview of Published Articles

Motor competence is a fundamental component of children’s physical development, supporting their ability to participate in physical activity and contributing to health across the lifespan. The cross-sectional study by Pelemiš et al. (contribution 1) investigated the relationships between body composition and motor coordination performance in preschool children aged 6–7 years, while also examining potential sex differences. The authors found that among boys, body composition variables were significantly associated with performance in specific motor coordination tasks, including single-leg jumps and lateral jumps, whereas no significant associations were observed among girls. Although no sex differences were identified in body composition, girls achieved higher scores in selected motor coordination tasks (i.e., single-leg jumps), suggesting that the interaction between body composition and motor competence may differ according to sex during early childhood.

Complementing this perspective, Đurović et al. (contribution 2) investigated differences in motor coordination according to sex and participation in organized physical activity among Serbian preschool children aged 5.57 ± 0.49 years. Participants were grouped according to whether they engaged in additional organized physical activity beyond the regular kindergarten curriculum. Children participating in additional organized physical activity achieved higher scores across the motor coordination tests than those who did not participate in such activities. Also, results demonstrated that girls achieved higher scores than boys in walking backwards and in the overall motor quotient. Furthermore, age, participation in organized physical activity, and body mass index were associated with several motor coordination outcomes, suggesting that motor competence in early childhood is influenced by multiple interacting factors. Taken together, the studies by Pelemiš et al. and Đurović et al. provide complementary evidence on factors associated with motor coordination during early childhood, particularly body composition, participation in organized physical activity, and sex.

Body composition constitutes another important dimension of children’s health that warrants attention during preschool years. Reisberg et al. (contribution 3) examined the longitudinal relationships of MVPA, sedentary time (objectively measured), cardiorespiratory fitness, and muscular fitness, with body fatness during the transition from preschool to the first grade of school. After adjusting for confounding factors, the authors found that higher sedentary time and lower muscular fitness at preschool age were associated with higher body fatness in the first grade of school among girls, whereas no such associations were observed in boys. In contrast, neither MVPA nor cardiorespiratory fitness predicted subsequent body fatness in either sex. This study contributes to the limited longitudinal evidence on the relationships of movement behaviors and physical fitness with body fatness during the transition from preschool to primary school.

A broader perspective on child and adolescent health also requires attention to other modifiable lifestyle dimensions, including nutrition. Within this context, Sánchez-Díaz et al. (contribution 4) explored the associations between Mediterranean diet adherence, body mass index, and selected physical performance measures in 35 male youth handball players aged 15.6 ± 1.6 years. Using a clustering approach, the authors identified two distinct player profiles. Players characterized by higher adherence to the Mediterranean diet and lower body mass index also exhibited healthier dietary behaviors, including less frequent fast-food consumption, breakfast skipping, and consumption of sweets and candy, together with better performance in the 10 m sprint and countermovement jump. Given the cross-sectional design and small sample size, these findings should be interpreted cautiously. Nevertheless, the identified profiles may help generate hypotheses for future longitudinal and interventional research examining nutritional patterns, body composition, and physical performance in young athletes.

Extending the scope of the Special Issue to cognitive health, Cerda-Vega et al. (contribution 5) presented a systematic review protocol designed to evaluate the effects of physical exercise on executive function in children and adolescents with overweight or obesity. The review synthesized evidence from longitudinal intervention studies and examined the effects of different exercise characteristics on the core domains of executive function, including working memory, inhibitory control, and cognitive flexibility. By addressing important gaps identified in previous reviews, the protocol establishes a framework for a more comprehensive future synthesis of the available evidence, which may subsequently be incorporated into research and evidence-based practice in pediatric populations with overweight or obesity.

## 3. Concluding Remarks

The studies included in this Special Issue provide complementary evidence on different dimensions of child and adolescent health and development, including motor competence, physical activity, sedentary behavior, physical fitness, body composition, nutrition, and exercise. Together, these contributions illustrate the value of multidisciplinary approaches encompassing physical, behavioral, nutritional, and cognitive domains.

Importantly, the included studies address areas in which evidence remains limited, including factors associated with motor coordination in preschool children; the longitudinal relationships examined between physical activity, sedentary behavior, physical fitness, and body fatness; the associations between dietary profiles, body mass index, and physical performance in young athletes; and the planned synthesis of evidence on the effects of exercise on executive function in children and adolescents with overweight or obesity. Collectively, these contributions broaden current understanding of child and adolescent health and provide a basis for future research and health-promotion strategies.

Nevertheless, further longitudinal and interventional research involving larger and more diverse samples is needed to strengthen the available evidence and support more robust causal inference. In line with the recommendations emerging from the studies included in this Special Issue, future research should track changes in motor competence, physical activity, physical fitness, and body composition across developmental stages; incorporate objective measures of physical activity and broader assessments of motor competence; and, where relevant, use direct assessments of body composition and account for biological maturation. Further evidence is also needed to determine how exercise characteristics may influence specific domains of executive function in children and adolescents with overweight or obesity. By bringing together these complementary perspectives, this Special Issue provides a valuable foundation for advancing multidisciplinary research and informing strategies to promote health, development, and well-being throughout childhood and adolescence.

The contributions included in this Special Issue are also relevant to the United Nations Sustainable Development Goals, particularly SDG 3 (Good Health and Well-Being), by advancing knowledge on different dimensions of health and development during childhood and adolescence, and SDG 4 (Quality Education), by highlighting the relevance of preschool and school contexts for supporting motor development and active lifestyles. Although the included studies did not directly assess progress towards these goals, their findings and methodological contributions may inform multidisciplinary research and initiatives aimed at promoting health, development, and well-being from an early age.

## Data Availability

No new data were created or analyzed in this study. Data sharing is not applicable to this article.

## Abbreviations

The following abbreviations are used in this manuscript:

WHO	World Health Organization
MVPA	Moderate-to-vigorous physical activity
